# Land Planarian Assemblages in Protected Areas of the Interior Atlantic Forest: Implications for Conservation

**DOI:** 10.1371/journal.pone.0090513

**Published:** 2014-03-05

**Authors:** Lisandro Negrete, Karine D. Colpo, Francisco Brusa

**Affiliations:** 1 División Zoología Invertebrados, Facultad de Ciencias Naturales y Museo, Universidad Nacional de La Plata, La Plata, Buenos Aires, Argentina; 2 Laboratorio de Biología de la Reproducción y el Crecimiento de Crustáceos Decápodos. Departamento de Biodiversidad y Biología Experimental, Facultad de Ciencias Exactas y Naturales, Universidad de Buenos Aires, Buenos Aires, Argentina; 3 Consejo Nacional de Investigaciones Científicas y Técnicas; Estacion Experimental de Zonas Áridas (CSIC), Spain

## Abstract

Land planarians are an interesting group of free-living flatworms that can be useful as bioindicators because of their high sensitivity to environmental changes and low dispersal capacity. In this study, we describe and compare assemblages of land planarians from areas with different conservation degrees of the Interior Atlantic Forest (Misiones, Argentina), and assess factors that could be related to their abundance and richness. Eight sites were tracked in search of land planarians in Reserva de Vida Silvestre Urugua-í (RVSU) and Campo Anexo Manuel Belgrano (CAMB). Diurnal and nocturnal surveys were performed in each site along nine sampling campaigns. We collected 237 individuals belonging to 18 species of the subfamily Geoplaninae. All sites were dominated by *Geoplana* sp. 1 and *Pasipha hauseri*. The richness estimators showed that there would be more species in RVSU than in CAMB. The abundance and richness of land planarians was high during the night and after rainfalls, suggesting an increased activity of flatworms under such conditions. The abundance and richness of land planarians were also related to the conservation condition of the sites. Disturbed sites showed less abundance and richness, and were segregated from non-disturbed ones by nmMDS analysis. Beta diversity between sites was higher than expected, indicating that the species turnover between sites contributed more to the total richness (gamma diversity) than the alpha diversity.

## Introduction

Land planarians (Platyhelminthes: Geoplanidae) successfully colonized the terrestrial environment millions of years ago [1]. However, they have not been able to develop mechanisms for water conservation and are thus unable to withstand desiccation [2]. In addition, land planarians have low vagility and cannot endure long periods of immersion in water, so they can be considered stenoic organisms regarding their habitat requirements, i.e., they are very sensitive to the moisture conditions of the environment [2]. Land planarians are ‘top predators’ of the soil fauna [3], [4]. They can feed on a wide range of soil invertebrates, mainly earthworms, snails, lugs, leeches, insects, isopods, and arachnids [5]–[10]. Therefore, they may be good environmental indicators, particularly in tropical and subtropical rainforests, where they are abundant [3].

Land planarians exhibit the highest diversity in the Atlantic Forest, with about 180 species described for its Brazilian portion [11]. The Atlantic Forest is one of the world's 25 recognized biodiversity hotspots [12]. It is a complex of ecosystems which extends along the Atlantic coast of Brazil and inland as far as eastern Paraguay and north-eastern Argentina, constituting the Interior Atlantic Forest. The original coverage of the Atlantic Forest remains in small fragments and under some kind of conservation status [13], [14]. In Argentina, there are still large extensions of the original Atlantic Forest, mainly under some kind of legal protection. This ecosystem has been suffering from human impact that has modified the original landscape, due to uncontrolled deforestation (which has been the primary cause of forest degradation), the burning of the land to prepare it for farming or grazing, and the introduction of exotic species with commercial purposes [13].

Several researches have study the diversity of land planarians in the Atlantic Forest of Brazil [15]–[18], and some have compared different assemblages in man-disturbed areas [5], [19]. At present, the diversity of land planarians in the Argentine portion of the Atlantic Forest is unknown. Recently, we have started to describe the diversity of these flatworms in this region [20], [21]. In order to improve our knowledge about land planarians, in this work we describe and compare assemblages from areas of the Interior Atlantic Forest of Argentina with different conservation degrees, and assess factors that could be related to their abundance and richness. Additionally, we determine the contribution of species richness (alpha) and species turnover (beta) to the gamma diversity.

## Methods

### Ethics Statement

The research has been conducted according to the Argentine law.

### Study area and sampling design

Our study took place in the southern portion of the Interior Atlantic Forest. In Argentina, this region covers about 25,700 km^2^ and is part of the Paranaense Subregion (Neotropical Region), where two biogeographical provinces are recognized: the Paranaense Forest and the Moist Forest with the coniferous tree *Araucaria angustifolia* [22]. These provinces are characterized as semi-deciduous subtropical rainforest. The climate is warm and humid, with an annual temperature of 16–22°C and a total annual precipitation of 1,600–2,000 mm [23].

**Figure 1:**
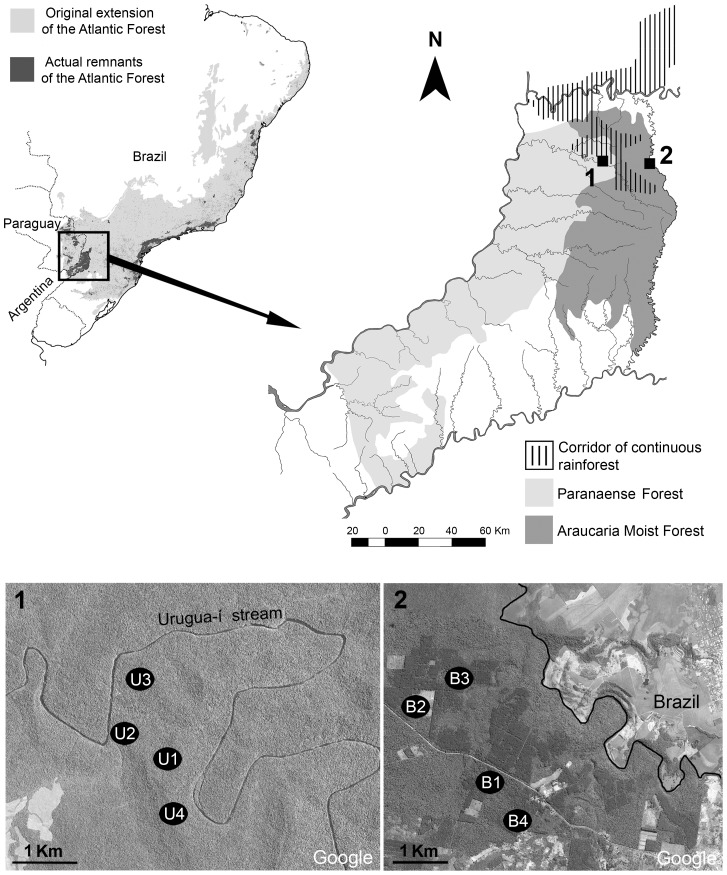
Study area, in the Argentine portion of the Interior Atlantic Forest (Misiones province). 1, Reserva de Vida Silvestre Urugua-í (RVSU), with four undisturbed sampling sites (U1–U4), and 2, Campo Anexo Manuel Belgrano (CAMB), with two undisturbed sampling sites (B1 and B3), and two disturbed sampling sites (B2 and B4). Maps modified from Di Bitetti et al. (2003), Galindo-Leal and Câmara (2003), and Google Earth.

The surveys were performed in two reserves of Misiones province, separated by about 40 km: Reserva de Vida Silvestre Urugua-í (RVSU) (25° 59′ S, 54° 05′ W) and Campo Anexo Manuel Belgrano (CAMB) (26° 02′ S, 53° 47′ W), each representing the Paranaense Forest and Moist Forest with *A. angustifolia*, respectively (Figure 1). The reserves are differentiated by conserved surface, altitude, management degree, and vegetation type. RVSU is a private natural reserve that covers 3,423 ha at ∼200 m a.s.l. It was created in 1997 and previously used for selective logging until the 1970s. This reserve, now under strict protection, is part of one of the largest corridors of continuous original rainforest in the southern portion of the Atlantic Forest, a ‘green block’ of almost 6,000 km^2^ [24]. It is characterized by diversified forests, although trees of *Balfourodendron riedelianum* and *Nectandra* spp. dominate plant formations. CAMB is a governmental forest reserve that covers 2,136 ha at ∼600 m a.s.l. It was created in 1948 to protect native and planted populations of *A. angustifolia*. This rainforest is also characterized by an undergrowth of tree ferns (*Alsophyla* sp., *Dicksonia* sp., *Trichipteris* sp.) [24]. However, in CAMB there are also plantations with exotic conifers (*Pinus taeda*). Therefore, this reserve is a mosaic of preserved and disturbed areas, isolated from other protected areas and surrounded mainly by small farms (Figure 1).

In each reserve, four sites were tracked in search of land planarians. All sites were selected for their accessibility, using existing paths. In RVSU, the four sites (U1–U4) were located in undisturbed areas with a condition of native vegetation and with the same physiognomy. In CAMB, the four sites (B1–B4) were situated in heterogeneous land use areas. Sites B1 and B3 were situated in undisturbed areas with *A. angustifolia*, while B2 and B4 were located in disturbed areas with exotic vegetation (*P. taeda*) (Figure 1).

In both reserves, land planarians were collected with the same sampling procedure along nine campaigns, between 2008 and 2010. In each site, samplings were performed by one person walking along the paths (1,000–1,500 m long) for 2 h during the day and another 2 h during the night. Therefore, 36 hours of sampling (2 h diurnal ×2 h nocturnal ×9 campaigns) were carried out in each site, totaling 144 hours of sampling effort for each reserve. During the day, the collector searched for planarians beneath and inside fallen logs, leaf litter and stones, whereas during the night, when planarians are more active, the collector performed the direct observation of the soil by means of a headlamp. Land planarians were manually collected and placed in plastic recipients with humid leaf litter, to avoid dehydration stress.

Environmental data were obtained from climatological stations near the reserves. The mean temperature of each sampling day and the cumulative rainfall (sum of millimeters of the ten days previous to each sampling day) were recorded.

### Morphological analysis

The external features of each specimen were observed in live. Planarians were then killed by throwing boiling water on them to avoid distortions of their tissues and then fixed in 10% formaldehyde and preserved in 70% ethanol. Fragments of different body regions (anterior region, pre-pharyngeal region, pharynx and copulatory apparatus) were dehydrated and embedded in Paraplast, serially sectioned at 6–10 µm thick with a microtome, and stained with hematoxylin-eosin and the Masson's trichrome method [25]. The histological preparations were observed by optical microscope and the copulatory apparatus reconstructed for identification purposes. The specimens were studied by two specialists on free living flatworms (LN and FB). Some specimens were identified at species level and others as morphospecies because they are in description process.

The material studied was deposited in the Invertebrate Collection at Museo de La Plata (MLP), Argentina.

### Data analysis

Species richness is the simplest way to describe the diversity of a community and to make comparisons [26]. We disaggregated species richness in (a) punctual alpha diversity, for the number of land planarian species recorded in each site, and (b) cumulative alpha diversity, for the total number of species in each reserve. We constructed species accumulation curves using sample-based rarefaction, with the expected richness function Mau Tau (with 95% confidence intervals), to compare cumulative alpha diversity curves between reserves [27]. We used the species accumulation curves with the number of individuals instead of the number of samples to avoid biases in comparison due to differences in the abundance of land planarians [28]. Since all inventories have unrecorded species [29], to analyze and compare the completeness of the species inventory in each reserve, we tested the performance of eight richness estimators, based on abundance (Chao 1, ACE) and incidence (Chao 2, ICE, jacknife 1, jacknife 2, bootstrap, and Michaelis-Menten), and the number of singletons and doubletons. Species accumulation curves and richness estimators were computed using Estimates v.9.0 [30], performing 100 randomizations in each analysis. To analyze the dissimilarity among reserves, we performed the calculation as 1 – Bray-Curtis index, using Chao's procedure, with Estimates v.9.0, because it compensates the effect of unseen shared species [31].

Abundance and species richness are interesting parameters to assess the structure of assemblages, since they are simple and fast to measure. Furthermore, these parameters can change according to environmental conditions. Besides the effects of mean temperature and cumulative rainfall on abundance and richness of land planarians, we also evaluated the relationship of these parameters with the conservation condition of each site. Therefore, we performed a multiple regression analysis (GLM), including data of both reserves (2 reserves ×4 sites ×9 campaigns), to test the effects of three independent variables: mean temperature, cumulative rainfall (continuous predictor variables), and conservation condition (categorical predictor variable: disturbed/undisturbed) on two dependent variables (abundance and richness).

To assess whether we had found more land planarians in diurnal or nocturnal surveys, we used a non-parametric Wilcoxon test, for dependent samples. We compared the abundance of planarians collected in 36 diurnal and nocturnal samplings (4 sites ×9 campaigns) in each reserve. After this comparison, we pooled diurnal and nocturnal sampling data, summarizing nine replicates (campaigns) per site. This procedure ensured that the actual richness and abundance of each site was well estimated.

We then used a two-way ANOVA to tested the effect of the reserves (fixed, orthogonal factor, with two levels: RVSU and CAMB) and sites (random, nested factor, with U1–U4 nested in RVSU level, and B1–B4 nested in CAMB level) on the abundance and richness of land planarians. Data were log-transformed to fulfill ANOVA assumptions. Post-hoc Student-Newman-Keuls (SNK) tests were applied for multiple comparisons.

We built rank-abundance (dominance-diversity) curves as indicators of the structure of the planarian assemblages of each site in both reserves. The relative abundance of each taxon on a logarithmic scale (log_10_) was plotted against the rank order of the taxa, from the most to the least abundant. These curves constitute a useful tool to visualize some aspects of the assemblages such as species richness (number of points), evenness (slope), number of rare species (tail of curves) and relative abundance of each species (order of the species in curves) [32]. The composition of land planarian assemblages was compared among sites of RVSU and CAMB by the non-metric multidimensional scaling (nmMDS), using the Bray-Curtis coefficient, to evaluate the similarities based on the abundance matrix (log-transformed).

Additionally, we determined the degree of contribution of species richness (alpha) and species turnover (beta) between sites to landscape scale diversity (gamma diversity). We adopted the framework of Jost [33], [34] to calculate alpha (diversity within samples) and beta (diversity among samples), in which gamma diversity (total amount of diversity) is partitioned according to the formula H_α_ *H_β_ = H_γ_ (H: Shannon-Wiener entropy). Entropies, like the Shannon-Wiener index, are not themselves diversities, and their use may obscure differences in diversity because indices differ only by small magnitudes [35]. Therefore, we used a transformation that allows an intuitive interpretation of species diversity by using the effective number of species, named *^q^D* by Jost [33], as a measure of “true diversity”. We computed the diversity with *q* = 0, where diversity is completely insensitive to the abundances of species and the value obtained is thus equivalent to species richness (*^0^D* = S_obs_), and *q* = 1, the exponential Shannon entropy, where each species is included with a weight proportional to its abundance (*^1^D* = exp H′) [36]. We used Partition v.3.0 [37], using 1000 randomizations (Monte Carlo resampling method) to derive the expected values of alpha and beta diversity that would be obtained if individuals or samples were randomly distributed.

## Results

**Figure 2:**
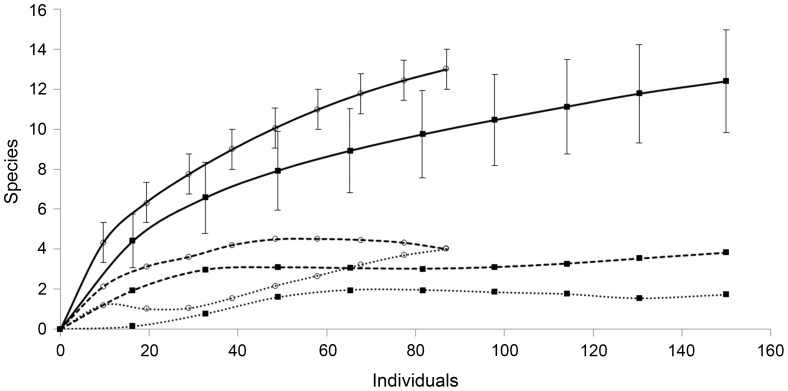
Land planarian species rarefaction curves, singleton and doubleton curves for reserves. Solid lines show the rarefaction curves (bars delineate 95% confidence intervals). Dashed and dotted lines represent the singleton and doubleton curves, respectively. RVSU (squares) and CAMB (circles).

We found 237 individuals (150 in RVSU and 87 in CAMB), representing 18 land planarian species distributed in six genera of the subfamily Geoplaninae. We found 12 species in RVSU and 13 species in CAMB (Table 1). For cumulative alpha diversity, the species rarefaction curves were not different between reserves (Figure 2). However, singleton and doubleton curves showed different trends. In CAMB but not in RVSU, singleton and doubleton curves reached the intersection with each other (Figure 2). Among the richness estimators analyzed, bootstrap was the most conservative one, while Chao 2 estimated the highest number of species in RVSU (Table 2). In CAMB, Chao 1 and bootstrap estimated the lowest number of species, similar to that recorded, whereas ICE estimated the highest number of species. On average, taking into account the eight estimators, we reached 65% of completeness of the inventory of species for RVSU and 77% of that for CAMB (Table 2). Five species were unique to RVSU, whereas six species were unique to CAMB, being seven species shared by both reserves (Table 1). According to Chao estimation, we found a dissimilarity of 38% between reserves (Bray-Curtis similarity index = 0.62).

**Table 1:** Abundance and richness (alpha diversity) of land planarian species collected in each sampling site. Letters (A–R) identify the species in Figure 4.

Reserves		RVSU					CAMB					
Taxa/Sampling sites		U1	U2	U3	U4	Total	B1	B2	B3	B4	Total	
*Choeradoplana crassiphalla*	A	3	-	-	5	8	-	-	2	1	3	11
*Geoplana* sp. 1	B	18	38	7	12	75	10	1	13	3	27	102
*Geoplana* sp. 2	C	1	-	-	6	7	4	-	7	1	12	19
*Geoplana* sp. 3	D	-	-	-	-	-	-	-	2	-	2	2
*Geoplana* sp. 4	E	-	-	1	-	1	-	-	2	-	2	3
*Geoplana* sp. 5	F	-	-	-	2	2	-	-	-	-	-	2
*Geoplana* sp. 6	G	-	-	-	-	-	1	-	1	-	2	2
*Geoplana* sp. 7	H	-	-	-	-	-	-	-	1	-	1	1
*Gigantea* sp. 1	I	-	1	-	-	1	-	-	-	-	-	1
*Matuxia* cf. *matuta*	J	-	-	-	-	-	1	-	-	-	1	1
*Pasipha hauseri*	K	31	5	2	4	42	11	9	10	-	30	72
*Pasipha* sp. 1	L	1	-	-	-	1	-	-	-	-	-	1
*Pasipha* sp. 2	M	-	-	-	3	3	-	-	-	-	-	3
*Pasipha* sp. 3	N	2	-	-	-	2	-	-	-	-	-	2
*Pasipha* sp. 4	O	-	-	-	-	-	-	-	1	-	1	1
*Pasipha* sp. 5	P	-	-	-	-	-	-	-	1	-	1	1
*Supramontana argentina*	Q	1	1	-	1	3	-	-	2	-	2	5
*Xerapoa* cf. *pseudorhynchodemus*	R	2	1	2	-	5	2	-	-	1	3	8
Total abundance		59	46	12	33	150	29	10	42	6	87	237
Punctual alpha diversity		8	5	4	7		6	2	11	4		
Cumulative alpha diversity		12					13					

**Table 2:** Land planarian richness, singletons and doubletons observed in each reserve.

	RVSU		CAMB	
Cumulative alpha diversity	12		13	
Number of singletons	4		4	
Number of doubletons	2		4	
Chao 1	17	71%	15	87%
ACE	16	75%	16	81%
Chao 2	28	43%	16	81%
ICE	19	63%	20	65%
Jacknife 1	17	71%	17	76%
Jacknife 2	21	57%	19	68%
Bootstrap	15	80%	15	87%
Michaelis Menten	19	63%	19	68%
Average of inventory completeness		65%		77%

Number of species expected and percentages of inventory completeness according to different richness estimators.

**Figure 3:**
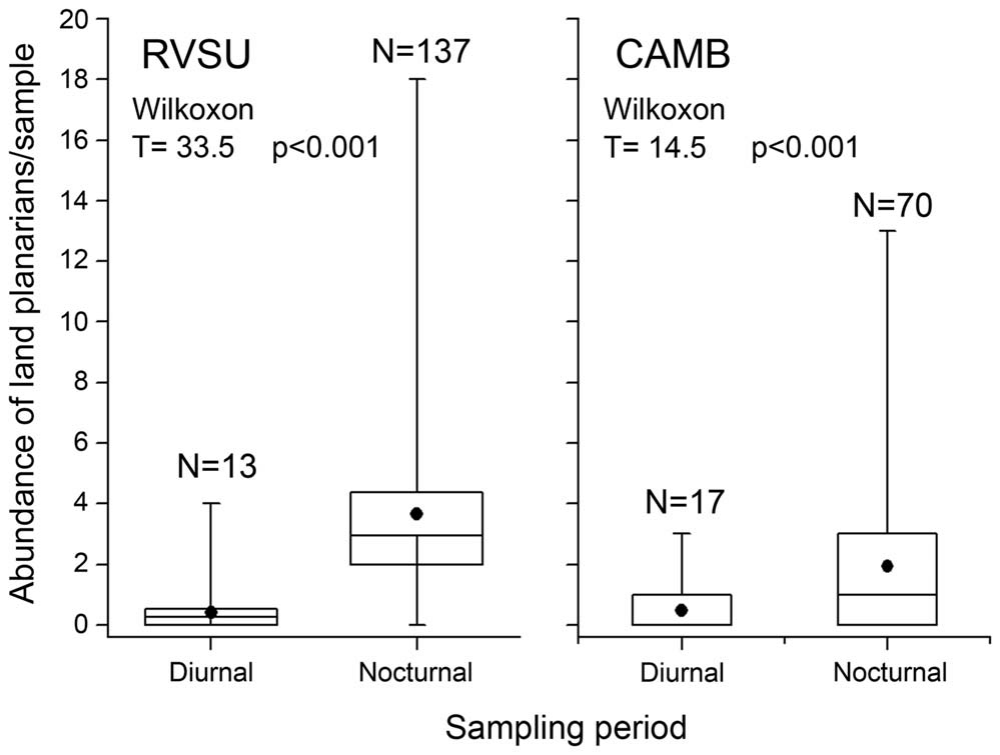
Comparison of land planarian abundance between 36 pairs of diurnal and nocturnal surveys, in each reserve (RVSU and CAMB). Non-parametric Wilcoxon test, for dependent samples was used. Mean (dot), median (line inside the box), 25–75% (box), min-max (whiskers), and N =  total abundance of land planarians.

During the study, the mean temperature varied between 9.6°C and 27.9°C, and the cumulative rainfall varied between 6.5 mm and 130 mm. The cumulative rainfall showed a positive effect on the abundance (p = 0.037) and richness (p = 0.020) of land planarians, while the temperature was not related. The conservation condition also affected the abundance and richness (Table 3). In both reserves, we found a greater abundance of land planarians during the nocturnal samplings (RVSU: T = 33.5, p<0.0001 and CAMB: T = 14.5, p<0.001) (Figure 3). Approximately 90% of the individuals collected in RVSU (N = 137) and 80% of those collected in CAMB (N = 70) were recorded during the nocturnal surveys.

**Table 3:** Summary results of a GLM - multiple regression analysis, testing the relationship between independent variables (continuous: mean temperature, cumulative rainfall; and categorical: conservation condition) and two dependent variables (abundance and richness of land planarians).

		Dependent variables					
		Abundance			Richness		
Independent variables	df	MS	F	p	MS	F	p
Mean temperature	1	2.67	0.184	0.669	2.53	1.123	0.293
Cumulative rainfall	1	65.15	4.493	0.037	12.75	5.653	0.020
Conservation condition	1	110.2	7.602	0.007	22.16	9.817	0.002
Error	68	14.5			2.25		

**Figure 4:**
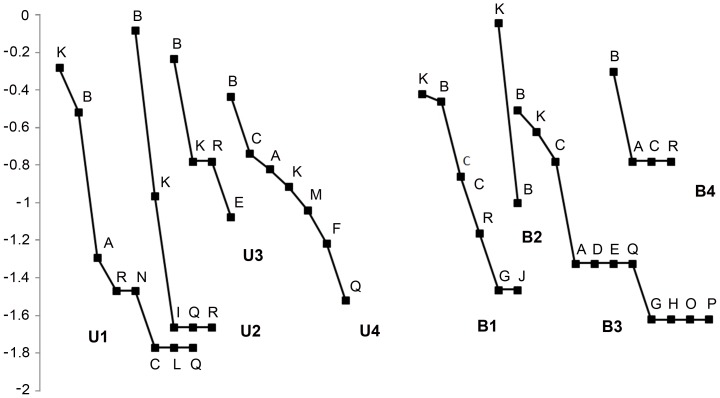
Rank-abundance curves of land planarian assemblages in each sampling site. Species codes are given in Table 1. *Geoplana* sp. 1 (B) and *Pasipha hauseri* (K) were the most abundant species in all sites.

*Geoplana* sp. 1 and *Pasipha hauseri* (Froehlich, 1959) together represented 74% of the total collected planarians (Table 1). *Geoplana* sp. 2, *Choeradoplana crassiphalla* (Negrete & Brusa, 2012) and *Xerapoa* cf. *pseudorhynchodemus* showed an intermediate level of abundance, totaling 16% of the land planarians collected. The remaining species showed low abundance (Table 1, Figure 4). The general abundance of land planarians varied from 12 to 59 individuals in RVSU, and from 6 to 42 in CAMB. In RVSU, the richness ranged from four species in U3 to eight species in U1. *Geoplana* sp. 1 was dominant in sites U2–U4 and *Pasipha hauseri* was dominant in U1. Only these two species were common to U1–U4. In CAMB, the number of species varied from two in B2 to 11 in B3. Only *Geoplana* sp. 1 was recorded in the four sites, being dominant in B3 and B4, and B1 together with *P. hauseri*, while the latter was dominant in B2 (Table 1, Figure 4).

**Figure 5:**
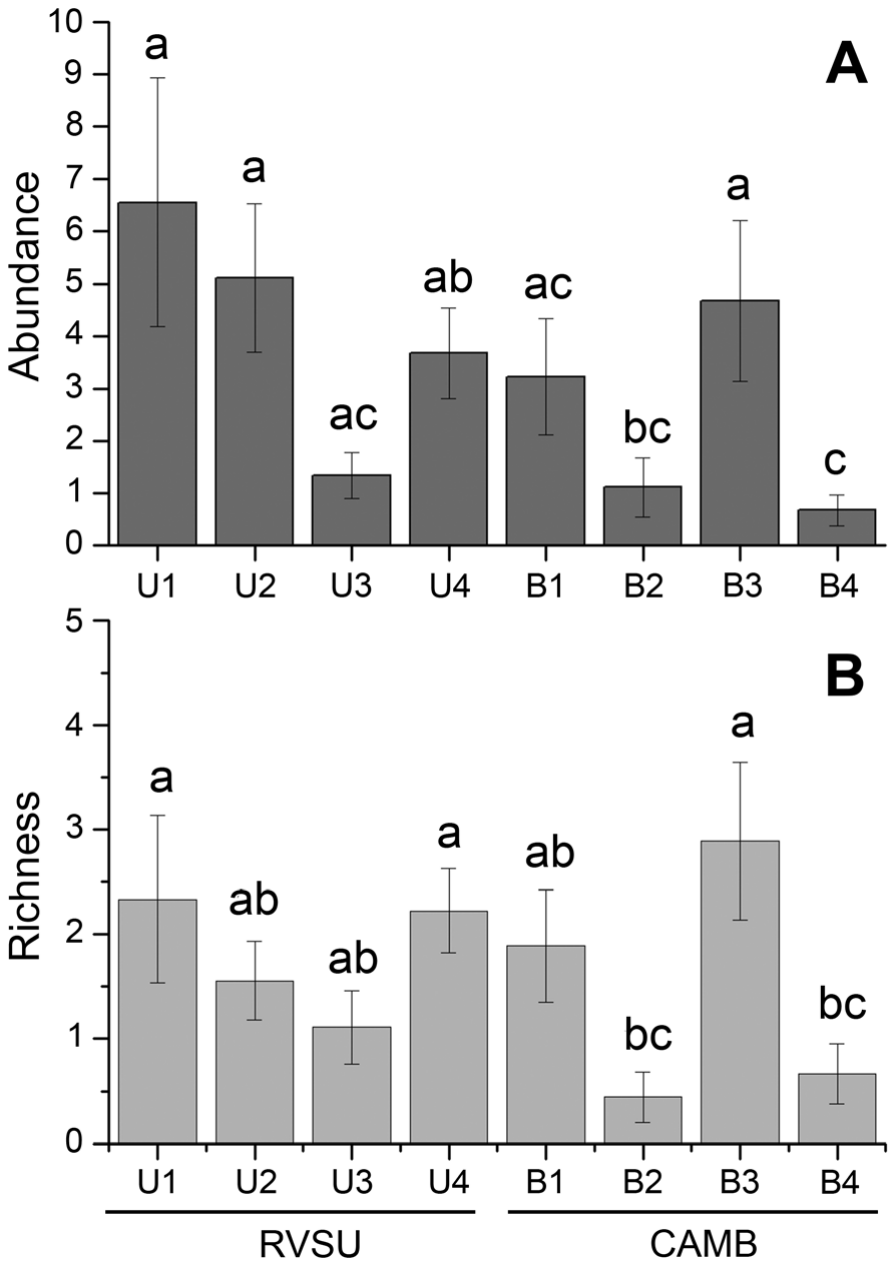
Comparison of abundance and richness of land planarians between sampling sites (U1–U4 and B1–B4). Means and standard errors. Different labels represent significant differences (Student-Newman-Keuls; α = 0.05).

**Figure 6:**
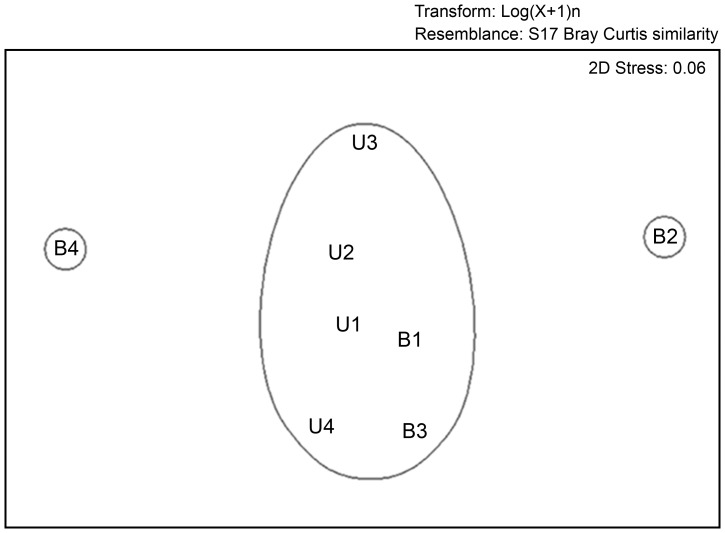
Non-metric multidimensional scaling (nmMDS) analysis based on the composition of land planarian assemblages of each sampling site. Sites with undisturbed condition (U1–U4, B1 and B3) were clustered at 54% similarity (Bray–Curtis coefficient), while disturbed sites B2 and B4 were segregated.

The reserves were not an important factor on the abundance (F = 1.789, p = 0.229) or on the richness (F = 0.789, p = 0.409) of land planarians. However, the abundance and the richness were sensitive to the different sites (Table 4). The disturbed sites B2 and B4 showed a lower abundance and richness than the undisturbed sites (U1–U4, B1 and B3) (Figure 5). Based on the composition of land planarian assemblages, the nmMDS analysis clustered sites with undisturbed condition at 54% similarity (Bray–Curtis coefficient). This analysis also segregated B2 and B4, which are disturbed sites (Figure 6).

**Table 4:** Summary results of a two-way ANOVA model, testing effects of the reserves (fixed; RVSU and CAMB) and sites (random, U1 to U4 nested in RVSU and B1 to B4 nested in CAMB) on the abundance and richness of land planarians.

			Abundance			Richness		
			C = 0.218 (NS)			C = 0.208 (NS)		
		df	MS	F	p	QM	F	p
Reserves	Fixed	1	0.681	1.789	0.229	0.154	0.789	0.409
Sites (Reserves)	Random	6	0.381	3.6	0.004	0.196	3.648	0.003
Error		64	0.106			0.054		

C = Cochran's test, NS  =  not significant.

Partitioning of the gamma diversity revealed that alpha species richness and alpha exponential Shannon diversity were not different from expected. However, beta diversity between sites was higher than expected for both measurements (Table 5).

**Table 5:** Partitioning of gamma diversity in alpha and beta components for species richness (*^0^D*) and exponential Shannon diversity (*^1^D*) compared to null models (expected values) calculated between sites.

	Species richness			D = exp H′		
Diversity	Observed	Expected	p	Observed	Expected	p
α	12.5	13.53 [11.5; 15]	0.907	2.03	2.23 [1.84; 2.34]	0.953
β	1.44	1.33 [1.2; 1.57]	0.015	1.23	1.05 [1; 1.27]	0.002
γ	18	17.99		2.49	2.34	

## Discussion

We observed no differences in cumulative species richness curves between reserves, and rising curves imply incomplete inventories. However, the singletons curve in CAMB reached the asymptote, tended to zero and intersected the doubletons curve, indicating that the species inventory in this reserve would be close to completeness [38]. The species richness estimators are consistent with this assumption. In contrast, in RVSU, the singleton and doubleton curves suggest that the species inventory is still far from being complete. The species richness estimators for RVSU were more variable than those for CAMB. With Chao 1, which is sensitive to rare species [39], we reached about 70% of the species inventory in RVSU and almost 90% in CAMB. Nevertheless, according to Chao 2, which takes into account presence/absence data, we reached about 40% of the species inventory in RVSU and over 80% in CAMB. Therefore, it would be expected that not observed-species are recorded in new surveys. Other contributions on land planarian diversity did not reach the completeness of the species inventory either. Carbayo et al. [5], using Chao 1, and Leal-Zanchet et al. [15], using Chao 2, obtained 60% of the species inventory for different land planarian assemblages. Considering that RVSU is part of the largest corridor of continuous preserved rainforest, it would be expected that this area would have higher species richness than CAMB. However, we found similar richness in both reserves, but according to Chao 2, which is unbiased for small samples [38], 16 species were not recorded in RVSU and only three species were not recorded in CAMB.

Studying different land planarian assemblages, Antunes et al. [16] recorded a similarity of ∼10% in species composition among two peri-urban forest areas, distant each other by about 60 kilometers. Baptista et al. [40] recorded 30% of similarity between two land planarian assemblages separated by over 260 kilometers in southern Brazil. Compared to these results, the similarity estimated between RVSU and CAMB (∼60%) is rather high. This similarity can be a consequence of a short distance between reserves, and due to the fact that RVSU is located on the eastern limit of the Paranaense Forest, very close to the Moist Forest of *Araucaria*, which would allow the sharing of species.

The cumulative rainfall, but not the temperature, affected the abundance and richness of flatworms. It is probable that the high moisture available everywhere makes unnecessary for land planarians to retreat to refuges (e.g. under fallen logs, leaf litter, and stones) [41]. Most individuals in both reserves were collected during nocturnal samplings. Although the behavior of land planarians is still poorly understood, our findings may indicate an increased activity of planarians during the night and after rainfalls, which would explain the high abundance and richness recorded under such conditions. In other studies, in which the samplings were carried out only during the day, no relationship was observed between abundance and richness of land planarians with environmental conditions [15], [42].

The pattern of species abundances observed in both reserves, in which a few species are very abundant (*Geoplana* sp. 1 and *Pasipha hauseri*), others are moderately represented, and many are rare species, seems to be common for land planarian assemblages, in agreement with that previously reported [5], [17], [18], [43]. The high abundance of *Geoplana* sp. 1 and *P. hauseri* in all sites surveyed can be due to the fact that these species are generalists, with the ability to colonize undisturbed habitats and habitats with different disturbance degrees, being more tolerant or with better capacity to thrive in environments under different conditions. Moreover, we found these species in pine plantations and gardens in small towns close to the reserves. Similarly, other geoplanid species, such as *Choeradoplana iheringi* Graff, 1899, *Obama ladislavii* (Graff, 1899) and *Paraba franciscana* (Leal-Zanchet & Carbayo, 2001), which are the most abundant flatworms recorded in different assemblages of southern Brazil [5], [18], [19], [44], have been also found in preserved and man-disturbed areas, even close to human settlements, such as small gardens in cities and dump deposits [5], [44].

The abundance and richness of flatworms were related to the conservation condition of the sites. The lower abundance and richness in the disturbed sites (B2 and B4), is probably a consequence of the human intervention in these areas. Carbayo et al. [5] also found lower abundance of land planarians in areas reforested with *Pinus* sp. than in native forests. Baptista and Leal-Zanchet [17] recorded lower abundance of flatworms in forests under some kind of anthropogenic influence. Furthermore, the composition of assemblages of planarians of the undisturbed sites of CAMB (B1 and B3) was similar to that of the conserved sites of RVSU (U1–U4). These six sites possibly provide greater availability of microhabitats to be colonized by land planarians [42]. In sites B2 and B4, the factors that would limit the presence of land planarians are the low complexity of the forest, the low availability of refuges in the soil, and the high incidence of sunlight on the soil owing to the absence of forest strata and subsequent moisture loss and compaction of the soil [5], [19], [45].

The beta diversity between sites was higher than expected, indicating that the species turnover between sites contributed more to the total richness (gamma diversity) than the alpha diversity. The high beta diversity evidences that the sampling sites share few species [46], probably as a consequence of the particular characteristics of each site of RVSU and CAMB, which harbor different species of land planarians and restrict many of them to certain areas.

Our results suggest that land planarian assemblages are sensitive to the conservation degree of the rainforest. Our study also evidence that not only continuous areas with native forest (e.g., RVSU) but also fragmented landscapes in which the original rainforest is conserved as patches (e.g., B1 and B3 in CAMB) may harbor similar levels of land planarian diversity. Considering the high levels of beta diversity between sites and the low vagility of land flatworms, we suggest that conservation policies should promote the connectivity between fragmented areas.

## References

[1] Sluys R (1999) Global diversity of land planarians (Platyhelminthes, Tricladida, Terricola): a new indicator-taxon in biodiversity and conservation studies. Biodivers Conserv 8: 1663–1681.

[2] Kawaguti S (1932) On the physiology of land planarians. Mem Fac Sci Agr Taihoku Imp Univ 7: 15–55.

[3] Sluys R (1998) Land planarians (Platyhelminthes, Tricladida, Terricola) in bio-diversity and conservation studies. Pedobiologia 42: 490–494.

[4] Estes JA, Terborgh J, Brashares JS, Power ME, Berger J, et al (2011) Trophic downgrading of planet Earth. Science 333: 301–306. 21764740 10.1126/science.1205106

[5] Carbayo F, Leal-Zanchet AM, Vieira EM (2002) Terrestrial flatworm (Platyhelminthes: Tricladida: Terricola) diversity versus man-induced disturbance in an ombrophilous forest in southern Brazil. Biodivers Conserv 11: 1091–1104.

[6] Ogren RE (1995) Predation behaviour of land planarians. Hydrobiologia 305: 105–111.

[7] Prasniski MET, Leal-Zanchet AM (2009) Predatory behaviour of the land flatworm *Notogynaphallia abundans* (Platyhelminthes: Tricladida). Zoologia 26: 606–612.

[8] Terrace TE, Baker GH (1994) The blue land planarian, *Caenoplana coerulea* Moseley (Tricladida: Geoplanidae), a predator of *Ommatoiulus moreleti* (Lucas) (Diplopoda: Julidae) in Southern Australia. Aust J Entomol 33: 371–372.

[9] Terrace TE, Baker GH (1996) Predation of earthworms by the land planarian, *Australoplana sanguinea* (Moseley) var. *alba* (Dendy) sensu Jones, 1981 (Tricladida: Geoplanidae). Tran R Soc S Aust 120: 177–178.

[10] Winsor L, Johns PM, Barker GM (2004) Terrestrial planarians (Platyhelminthes: Tricladida: Terricola) predaceous on terrestrial gastropods. In: Barker GM, editor. Natural enemies of terrestrial molluscs. Wallingford: CAB International.pp. 227–277.

[11] Carbayo F, Froehlich EM, Leal-Zanchet AM, Amato SB (2009) Turbelários (Platyhelminthes). In: Moreira da Rocha R, Pereira Boeger WA, editors. Estado da arte e perspectivas para a Zoologia no Brasil. Paraná: Editora da Universidade Federal do Paraná. pp. 49–64.

[12] Myers N, Mittermeier RA, Mittermeier CG, da Fonseca GAB, Kent J (2000) Biodiversity hotspots for conservation priorities. Nature 403: 853–858. 10706275 10.1038/35002501

[13] Galindo-Leal C, Câmara IG (2003) The Atlantic Forest of South America: biodiversity status, threats, and outlook. Washington DC: Center for Applied Biodiversity Science and Island Press. 489 p.

[14] Ribeiro MC, Metzger JP, Martensen AC, Ponzoni FJ, Hirota MM (2009) The Brazilian Atlantic Forest: How much is left, and how is the remaining forest distributed? Implications for conservation. Biol Conserv 142: 1141–1153.

[15] Leal-Zanchet AM, Baptista V, Miranda Campos L, Fraga Raffo J (2011) Spatial and temporal patterns of land flatworm assemblages in Brazilian Araucaria forests. Invertebr Biol 130: 25–33.

[16] Antunes MB, Marques DIL, Leal-Zanchet AM (2008) Composição das comunidades de planárias terrestres (Platyhelminthes, Tricladida, Terricola) em duas áreas de floresta estacional semidecidual do sul do Brasil. Neotrop Biol Conserv 3: 34–38.

[17] Baptista VA, Leal-Zanchet AM (2010) Land flatworm community structure in a subtropical deciduous forest in Southern Brazil. Belg J Zool 140: 83–90.

[18] Fick IA, Leal-Zanchet AM, Vieira EM (2006) Community structure of land flatworms (Platyhelminthes, Terricola): comparisons between Araucaria and Atlantic forest in Southern Brazil. Invertebr Biol 125: 306–313.

[19] Carbayo F, Leal-Zanchet AM, Vieira EM (2001) Land planarians (Platyhelminthes, Tricladida, Terricola) as indicators of man-induced disturbance in a South Brazilian rainforest. Belg J Zool 131: 223–224.

[20] Negrete L, Brusa F (2012) *Choeradoplana crassiphalla* sp. nov. (Platyhelminthes: Tricladida: Geoplanidae): a new species of land planarian from the Atlantic Forest of Argentina. Stud Neotrop Fauna Environ 47: 227–237.

[21] Negrete L, Leal-Zanchet AM, Brusa F (2014) A new species of *Supramontana* Carbayo & Leal-Zanchet (Platyhelminthes, Continenticola, Geoplanidae) from the Interior Atlantic Forest. Zootaxa 3753: 177–186. 24872289 10.11646/zootaxa.3753.2.7

[22] Morrone JJ (2001) Biogeografía de América Latina y el Caribe. Zaragosa: M&T Manuales y Tesis SEA. 148 p.

[23] Di Bitetti MS, Placci G, Dietz LA (2003) Una visión de biodiversidad para la Ecorregión del Bosque Atlántico del Alto Paraná: diseño de un paisaje para la conservación de la biodiversidad y prioridades para las acciones de conservación. Washington DC: World Wildlife Fund. 154 p.

[24] Giraudo AR, Povedano H, Belgrano MJ, Krauczuk E, Pardiñas U, et al.. (2003) Biodiversity Status of the Interior Atlantic Forest of Argentina. In: Galindo-Leal C, Câmara IG, editors. The Atlantic Forest of South America: biodiversity status, threats, and outlook. Washington DC: Center for Applied Biodiversity Science e Island Press. pp. 160–180.

[25] Romeis B (1989) Mikroskopische Technik. München: Urban und Schwarzenberg. 697 p.

[26] Magurran AE (2004) Measuring biological diversity. Oxford: Blackwell Science Ltd. 256 p.

[27] Colwell RK, Mao CX, Chang J (2004) Interpolating, extrapolating, and comparing incidence-based species accumulation curves. Ecology 85: 2717–2727.

[28] Gotelli NJ, Colwell RK (2001) Quantifying biodiversity: Procedures and pitfalls in the measurement and comparison of species richness. Ecol Lett 4: 379–391.

[29] Chao A, Hwang W, Chen Y, Kuo C (2000) Estimating the number of shared species in two communities. Stat Sin 10: 227–246.

[30] Colwell RK (2013) EstimateS: Statistical Estimation of species richness and shared species from samples. Version 9.0. Available at: http://purl.oclc.org/estimates (accessed 2013 Nov 28).

[31] Chao A, Chazdon RL, Colwell RK, Shen T-J (2005) A new statistical approach for assessing similarity of species composition with incidence and abundance data. Ecol Lett 8: 148–159.

[32] Feinsinger P (2001) Designing field studies for biodiversity conservation. Washington DC: Island Press. 212 p.

[33] Jost L (2006) Entropy and diversity. Oikos 113: 363–375.

[34] Jost L (2007) Partitioning diversity into independent alpha and beta components. Ecology 88: 2427–2439. 18027744 10.1890/06-1736.1

[35] Pfeiffer M, Mezger D (2012) Biodiversity assessment in incomplete inventories: leaf litter ant communities in several types of bornean rain forest. PloS ONE 7: 1–9. 10.1371/journal.pone.0040729 PMC3398027 22815799

[36] Moreno CE, Barragán F, Pineda E, Pavón NP (2011) Reanálisis de la diversidad alfa: alternativas para interpretar y comparar información sobre comunidades ecológicas. Rev Mex Biodiv 82: 1249–1261.

[37] Veech JA, Crist TO (2009) Partition: software for hierarchical partitioning of species diversity, version 3.0. Available at: http://www.users.muohio.edu/cristto/partition.htm (accessed 2013 Nov 28).

[38] Colwell RK, Coddington JA (1994) Estimating the extent of terrestrial biodiversity through extrapolation. Philos Trans R Soc Lond B Biol Sci 345: 101–118. 7972351 10.1098/rstb.1994.0091

[39] Colwell RK, Coddington JA (1996) Estimating terrestrial biodiversity through extrapolation. In: Hawksworth DL, editor. Biodiversity.Measurement and estimation. London: Chapman & Hall. pp. 101–118. 10.1098/rstb.1994.0091 7972351

[40] Baptista VA, Oliveira SM, Leal-Zanchet AM (2010) Inventário de planárias terrestres (Platyhelminthes, Tricladida) em remanescente de Floresta Estacional Decidual do Sul do Brasil. Biota Neotrop 10: 247–252.

[41] Froehlich CG (1955) On the biology of land planarians. Bol Fac Fil Ciênc Letr Zoologia 20: 263–271.

[42] Antunes MB, Leal-Zanchet AM, Fonseca CR (2012) Habitat structure, soil properties, and food availability do not predict terrestrial flatworms occurrence in Araucaria Forest sites. Pedobiologia 55: 25–31.

[43] Castro RA, Leal-Zanchet AM (2005) Composição de comunidades de planárias terrestres (Platyhelminthes) em áreas de floresta estacional e de campo na região central do Rio Grande do Sul, Brasil. Acta Biol Leopold 27: 147–150.

[44] Baptista VA, Matos LB, Fick IA, Leal-Zanchet AM (2006) Composição das comunidades de planárias terrestres (Platyhelminthes, Tricladida, Terricola) do Parque Nacional dos Aparados da Serra, Brasil. Iheringia Ser Zool 96: 293–297.

[45] Fonseca CR, Ganade G, Baldissera R, Becker CG, Boelter CR, et al (2009) Towards an ecologically-sustainable forestry in the Atlantic Forest. Biol Conserv 142: 1209–1219.

[46] Moreno CE (2001) Métodos para medir la biodiversidad. Zaragosa: M&T Manuales y Tesis SEA. 84 p.

